# Preparation of Reduction-Responsive Camptothecin Nanocapsules by Combining Nanoprecipitation and In Situ Polymerization for Anticancer Therapy

**DOI:** 10.3390/pharmaceutics10040173

**Published:** 2018-10-03

**Authors:** Xiao-Qing Song, Cheng Tao, Wei Li, Jie-Xin Wang, Yuan Le, Jian-Jun Zhang

**Affiliations:** 1 College of Chemical Engineering, Beijing University of Chemical Technology, Beijing 100029, China; 2015200066@mail.buct.edu.cn (X.-Q.S.); 2016200052@mail.buct.edu.cn (C.T.); 2016200053@mail.buct.edu.cn (W.L.); wangjx@mail.buct.edu.cn (J.-X.W.); 2 Beijing Advanced Innovation Center for Soft Matter Science and Engineering, Beijing University of Chemical Technology, Beijing 100029, China; 3 Research Center of the Ministry of Education for High Gravity Engineering and Technology, Beijing University of Chemical Technology, Beijing 100029, China

**Keywords:** reduction-responsive, nanocapsule, disulfide bond, in situ polymerization, anticancer therapy

## Abstract

Stimuli-responsive systems for controlled drug release have been extensively explored in recent years. In this work, we developed a reduction-responsive camptothecin (CPT) nanocapsule (CPT-NC) by combining nanoprecipitation and in situ polymerization using a polymerized surface ligand and a disulfide bond-containing crosslinker. Dissolution rate studies proved that the CPT-NCs have robust drug-release profiles in the presence of glutathione (GSH) owing to the division of the disulfide bond crosslinker which triggers the collapse of the polymer layer. Furthermore, the in vitro investigations demonstrated that the CPT-NCs exhibited a high-cellular uptake efficiency and cytotoxicity for cancer cells of squamous cell carcinoma (SCC-15). Our approach thus presents an effective intracellular drug delivery strategy for anticancer therapy.

## 1. Introduction

Over the past decades, polymer nanocarriers, including amphiphilic copolymer micelles/polymersomes, polyester-based polymer nanoparticles, and polymer nanogels/nanocapsules, have been extensively explored for cancer chemotherapy aimed at enhancing drug solubility in aqueous phase, prolonging in vivo blood circulation time, improving passive tumor targeting by the enhanced permeability and retention (EPR) effect, and reducing side effects [1,2,3,4,5]. Additionally, the particle size, composition structure, and surface properties of polymer nanocarriers can also be precisely fabricated by designing and synthesizing functional polymers which possess various chemical composition, molecular weight, and functional groups. Amphiphilic polymer micelles/polymersomes constitute some of the most important candidates and have often been used to deliver anticancer drugs with low water solubility by incorporating them into their hydrophobic cores [6,7,8,9,10]. However, these conventional polymer micelles are unstable in the circulating blood in vivo, thus resulting in serious side effects owing to the premature leakage of the loaded drugs [11,12]. Alternatively, polyester-based polymer nanoparticles such as polylactide (PLA) and poly(lactide-*co*-glycolide) (PLGA) have high drug capacity and excellent drug encapsulation efficiency. However, the applications of these nanoparticles are always limited by the slow drug release profiles and poor stability in water. To address this tissue, covalently crosslinking strategies have been utilized to fabricate novel polymer nanocarriers, referred to as polymer nanocapsules/nanogels [13,14,15,16]. In these polymer nanocapsules, the crosslinked zone cannot only retain their structural integrity, but also acts as a physical barrier for preventing premature release of the loaded drugs, and reduces drug side effects during blood circulation.

In situ polymerization is an effective method for modification of functional polymer layers on the surface of nanomaterials [17,18,19]. During the in situ polymerization process, the polymer layer is gradually grown on the materials, and results in the simplification of the purification procedures, increased conjugation yields, and unique structures [20]. Furthermore, by choosing and combining different functional monomers and crosslinkers, the polymer layer with desired functionality can be easily modified on the surface of nanomaterials.

To date, various stimuli-responsive components, which are responsive to acidity, redox substances, enzymes, and light, have been extensively utilized to design polymer nanocarriers, aiming at the enhancement of the drug delivery efficiency to the cytoplasm or nucleus of cancer cells [21,22,23,24,25]. Reduction-responsive polymer nanocarriers based on disulfide bonds hold great promise for anticancer drug delivery because the intracellular fluid of cancer cells has a high concentration of glutathione (GSH) reducing agents, which can effectively divide the disulfide bond and break the polymer structure, thus resulting in a quick release of the loaded drugs [26,27,28].

**Figure 1:**
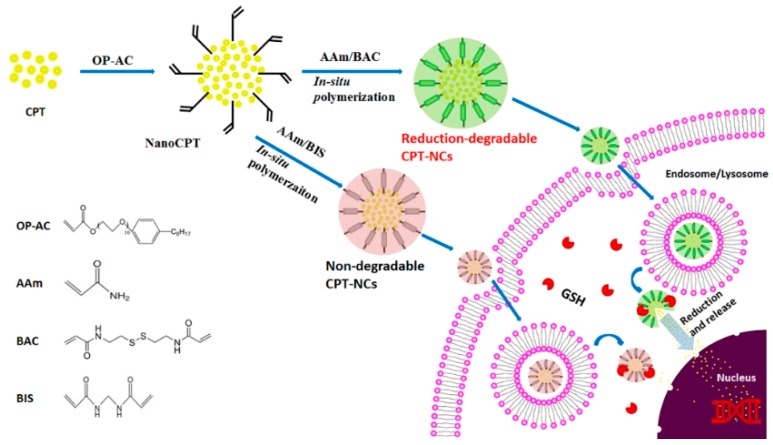
Schematic illustration showing the preparation and cellular uptake of the camptothecin nanocapsules (CPT-NCs). GSH: glutathione; BAC: *N*,*N*′-bis(acryloyl) cystamine; OP-AC: acrylated-alkylphenol ethoxylate; AAm: acrylamide; BIS: *N*,*N*′-methylene bisacrylamide.

Herein, we developed a reduction-responsive polymer drug nanocapsule by combining the nanoprecipitation technique, in situ polymerization, and a crosslinker that consisted of a disulfide bond. Camptothecin (CPT) was chosen as the model drug. It is an anticancer agent with a broad spectrum of activities for treating cancers of the liver, stomach, head, and neck, as well as leukemia, by selectively inhibiting deoxyribonucleic acid (DNA) topoisomerase [29]. A schematic illustration of the synthesis of the CPT nanocapsule (referred to as a CPT-NC) can be observed in Figure 1. Firstly, an amphiphilic polymerized acrylated-alkylphenol ethoxylate (OP-AC) molecule was used as the ligand for the preparation of CPT nanoparticles (NanoCPTs) with acrylate groups on its surface using the nanoprecipitation technique [30]. Subsequent in situ redox polymerization was executed using acrylamide (AAm) as the monomer, *N*,*N*′-methylene bisacrylamide (BIS) as the nondegradable crosslinker, and *N*,*N*′-bis(acryloyl) cystamine (BAC) as the reductive-degradable crosslinker, which resulted in both nonresponsive and reduction-responsive CPT-NCs, denoted as CPT-NCs-A and CPT-NCs-B, respectively. Furthermore, reduction-responsive CPT-NCs-B uptake can occur in cancer cells through the endocytosis pathway, and the polymer layer can be further degraded by an increased GSH concentration in the cytoplasm to promote the robust release of the loaded CPT. The released CPT will then diffuse into the cell nucleus and intercalate on the DNA to induce cell death. Moreover, as compared to CPT-NCs-A, in vitro investigations demonstrated that CPT-NCs-B exhibited a sensitive reduction-responsive drug release profile and an outstanding cytotoxicity against cancer cells in squamous cell carcinoma (SCC-15).

## 2. Materials and Methods

### 2.1. Materials

Camptothecin (CPT) was obtained from Beijing Zhongshuo Pharmaceutical Technology Development Co., Ltd. (Beijing, China) Additionally, *N*,*N*′-bis(acryloyl) cystamine (BAC) was purchased from Adamas-beta, (Shanghai, China), and 3-(4,5-dimethylthiazol-2-yl)-2,5-diphenyltetrazolium bromide (MTT) was obtained from Sigma-Aldrich (St. Louis, MO, USA). All other chemical reagents were purchased from the Shanghai Aladdin Chemistry Co., Ltd. (Shanghai, China) unless otherwise noted. All chemical reagents were of analytical grade or better, and were used without further purification.

### 2.2. Preparation and Characterization of the CPT-NCs

CPT was dissolved in dimethyl sulfoxide (DMSO) using sonification at a concentration of 2 mg/mL, and acted as the solvent phase. Additionally, OP-AC was dispersed in 10 mM sodium bicarbonate buffer with a pH = 8.5 and a concentration of 0.5 mg/mL, and acted as the antisolvent phase. A volume of 0.5 mL of the solvent phase was added to 10 mL of the antisolvent phase during stirring at room temperature. After stirring for 20 min, the NanoCPT dispersion was obtained after dialysis with phosphate-buffered saline (PBS, 15 mM, pH = 7.4) using a dialysis bag (MWCO = 3500 Da). Furthermore, the acrylamide monomer (AAm) was added during stirring. Subsequently, different crosslinkers were added, namely, *N*,*N*′-methylene bisacrylamide (BIS) for nondegradable CPT-NCs-A and *N*,*N*′-bis(acryloyl) cystamine (BAC) for degradable CPT-NCs-B. The molar ratio of monomer/crosslinker was adjusted to 5:2. The in situ redox polymerization was immediately initiated by adding 17 μL of *N*,*N*,*N*′,*N*′-tetramethylethylenediamine (TEMED) and 100 μL of ammonium persulfate (APS, 100 mg/mL, dissolved in deionized water). After polymerization for 90 min, the ultimate products were obtained using lyophilization after dialysis with PBS. These were stored at 4 °C for further use.

The morphologies of NanoCPTs and CPT-NCs were characterized by transmission electron microscopy (TEM, HT7700, Hitachi, Tokyo, Japan). The sample was prepared by adding a small droplet of dilute NanoCPT or CPT-NC dispersions on the copper grid with 300 mesh support carbon membrane, and then placed in a vacuum overnight. Hydrodynamic sizes and zeta potentials were measured by dynamic light scattering (DLS) using a Malvern Zetasizer Nano ZS90 instrument (Malvern Panalytical Ltd., Malvern, UK).

The content of CPT in CPT-NCs was determined using an ultraviolet-visible (UV-VIS) spectrophotometer (Varian Cary 50 ultraviolet-visible spectrophotometer, Walnut Creek, CA, USA) with an absorption wavelength of 368 nm according to the standard curve. The CPT loading capacity (*LC*) was calculated according to the following equation:

(1) $$LC\;\left(\%\right)=\frac{M_{L}}{M_{N}}\times 100\%$$

where *M_L_* is the mass of the encapsulated CPT, and *M_N_* is the mass of CPT-NCs. The *LC* was estimated to be 1.32% and 1.19% for CPT-NCs-A and CPT-NCs-B, respectively.

### 2.3. In Vitro CPT Release from the CPT-NCs

The in vitro drug release of CPT-NCs was analyzed using a dialysis method. In brief, 2 mg amounts of CPT-NCs-A and CPT-NCs-B were respectively dispersed in 5 mL of PBS buffer (pH = 7.4) at various concentrations of GSH (0 mM, 10 mM, and 20 mM). Additionally, the solution was placed into a dialysis bag (MWCO = 3500 Da), which was submerged into PBS buffer (10 mL, pH = 7.4) at the same GSH concentration. The release experiments were performed in a water bath at 37 °C with continuous stirring. At predetermined time points, 3 mL of the external buffer was removed to estimate the amount of drug released by the UV-VIS spectrophotometer at 368 nm, while the same amount of fresh buffer was added to keep the volume of the release medium invariable. All three measurements of the CPT release data were averaged.

### 2.4. In Vitro Cellular Uptake of the CPT-NCs

Qualitative cellular uptake of CPT-NCs was observed using a Leica TCS SP5 confocal laser scanning microscope (CLSM, Leica Microsystems, Wetzlar, Germany). Briefly, SCC-15 cells were seeded into a Lab-Tek™ chambered coverglass systems (8-wells) (Thermo Scientific Nunc Lab-Tek, Atlanta, GA, USA) at a density of 8 × 10^3^ cells/well in 200 µL of dulbecco’s modified eagle medium (DMEM) medium, and cultured overnight at 37 °C in 5% CO_2_ before use. Furthermore, 40 μL of free CPT, CPT-NCs-A, and CPT-NCs-B were added to a final CPT concentration of 840 ng/mL. After 4 h of incubation, the medium was removed and the cells were washed with PBS three times, followed by fixing with 4% paraformaldehyde at room temperature for 15 min. The cells were then washed three times with PBS and observed using a CLSM. The excitation and reception wavelengths of the CPT were 405 nm and 430 nm, respectively.

### 2.5. Intracellular Trafficking of the CPT-NCs

To determine the internalization pathway of CPT-NCs, SCC-15 cells (8 × 10^3^ cells per well) were seeded into an 8-well coverglass in 200 µL of medium and cultured for approximately 12 h. Cells were then exposed to free CPT, CPT-NCs-A, and CPT-NCs-B, at the final CPT concentration of 840 ng/mL. After incubation for 4 h or 24 h, the cells were stained with LysoTracker Green DND–26 (Wobisen, Beijing, China) for 2 h at 37 °C, followed by two washes with PBS. Finally, a CLSM was used to visualize the distribution of CPT-NCs. Excitation and emission occurred at 405 nm and 430 nm, respectively, while the LysoTracker Green DND–26 was excited at 504 nm with an emission at 511 nm.

### 2.6. In Vitro Cytotoxicity Assay

The induced cytotoxicity owing to free CPT and CPT-NCs in SCC-15 cells was determined using the MTT assay. The cells were seeded onto 96-well plates at a density of 4 × 10^3^ cells per well in 100 μL of medium and were incubated overnight. Free CPT, CPT-NCs-A, and CPT-NCs-B were diluted at various concentrations with medium. The sample medium solution (100 μL) was used to replace the medium in each well, and the plates were incubated for another 24 h. Subsequently, each sample solution was substituted with 100 μL of fresh medium and 20 μL of MTT solution (5 mg/mL in PBS). After 4 h of incubation, the medium solution was removed and 100 μL of DMSO was added to each well to dissolve the purple formazan crystals internalized by live cells. Finally, the absorbance was monitored at 570 nm (optical density (OD)_570nm_) using a microplate reader (Thermo Fisher, MK3, Waltham, MA, USA). In addition, cell viability was calculated according to the formula,

(2) $$\mathrm{Cell}\;\mathrm{viability}\;\left(\%\right)=\frac{{\mathrm{OD}}_{\frac{570\mathrm{nm}}{\mathrm{sample}}}}{{\mathrm{OD}}_{\frac{570\mathrm{nm}}{\mathrm{control}}}}\times 100\%$$

### 2.7. In Vitro Live/Dead Assay

SCC-15 cells were seeded into an 8-well coverglass at a density of 8 × 10^3^ cells/well in 200 µL of medium and were cultured to the appropriate confluence. Subsequently, 40 μL of free CPT, CPT-NCs-A, and CPT-NCs-B were added to reach a final CPT concentration of 840 ng/mL. After 24 h of culture, the cells were stained with a live/dead viability/cytotoxicity kit (Life technologies, Carlsbad, CA, USA), and were incubated at 37 °C for another 30 min. The cells were then washed once with PBS, followed by imaging using a CLSM.

### 2.8. In Vitro Apoptosis Assay

Drug-induced cell apoptosis was analyzed using the Annexin V-fluorescein isothiocyanate isomer (FITC)/propidium iodide (PI) apoptosis detection kit (Beyotime, Shanghai, China) using a CLSM and a MoFlo XDP flow cytometer (FCM, Beckman, Brea, CA, USA). For CLSM, SCC-15 cells were seeded into an 8-well coverglass with a density of 8 × 10^3^ cells/well in 200 µL of medium, and were cultured for approximately 12 h. Subsequently, the cells were respectively treated with free CPT, CPT-NCs-A, and CPT-NCs-B (final CPT concentration of 840 ng/mL) for 24 h. Subsequently, the cells were stained with Annexin V-FITC and PI and were observed using the CLSM. For FCM, SCC-15 cells were seeded in 6-well plates at a density of 1 × 10^5^ cells per well in 2 mL of medium and were cultured at 37 °C. Furthermore, the cells were respectively treated with PBS, free CPT, CPT-NCs-A, and CPT-NCs-B, at the final CPT concentration of 840 ng/mL for 24 h. The subsequent procedures were performed according to the manufacturer’s suggested procedures, and were detected using the FCM.

### 2.9. Statistical Analysis 

All statistical analyses were performed via Instat (GraphPad, San Diego, CA, USA) and SPSS 20.0 (Chicago, IL, USA). Experiments were statistically analyzed using the one-way ANOVA test using a 95% confidence interval.

## 3. Results and Discussion

### 3.1. Preparation and Characterization of the CPT-NCs

**Figure 2:**
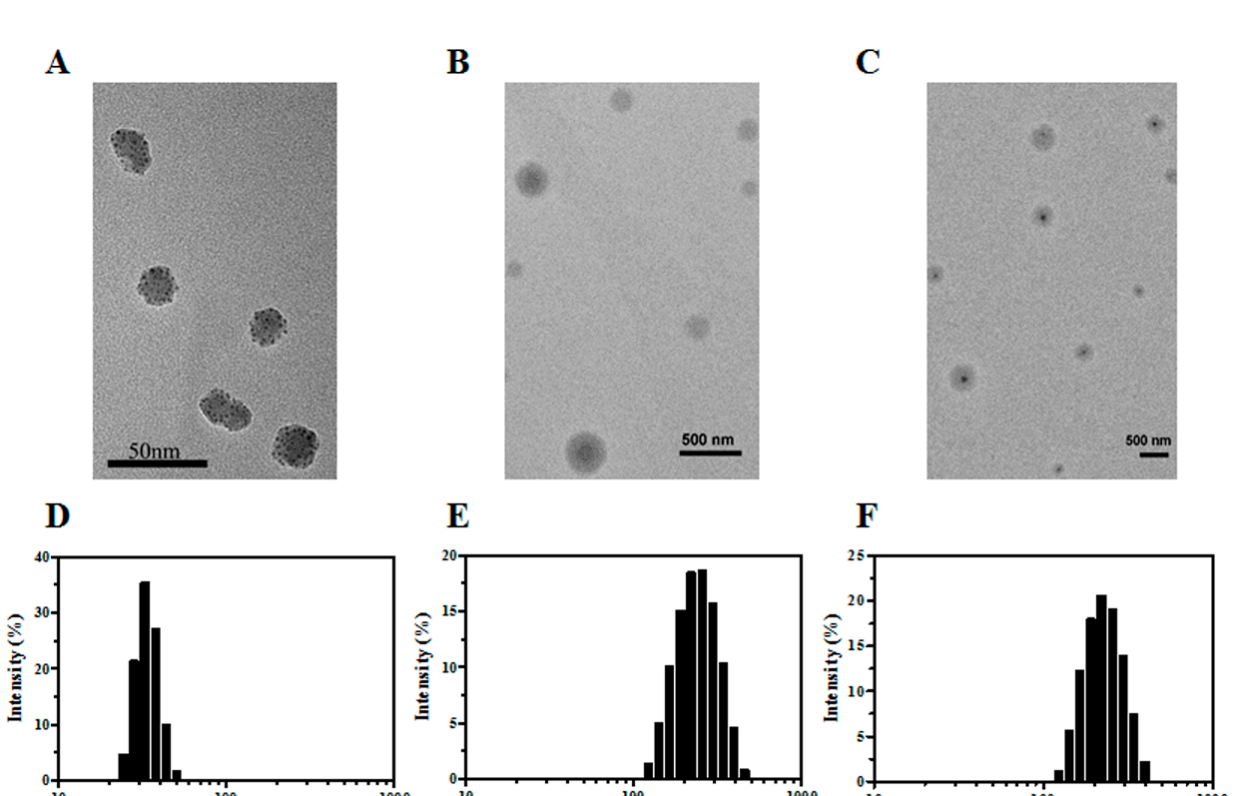
TEM images of (**A**) NanoCPTs; (**B**) CPT-NCs-A; (**C**) CPT-NCs-B; (**D**) Particle size distribution of NanoCPT; (**E**) CPT-NCs-A and (**F**) CPT-NCs-B. NanoCPTs: CPT nanoparticles; CPT-NCs-A: nonresponsive CPT-NCs; CPT-NCs-B: responsive CPT-NCs.

We first prepared NanoCPTs using the nanoprecipitation technique with OP-AC as the surface modification ligand. The TEM image showed that NanoCPTs had a spherical structure with particle sizes in the range of 30–40 nm that were well dispersed (Figure 2A). Furthermore, an average size of approximately 32.7 nm was determined based on DLS measurements (Figure 2D), which was consistent with TEM results. Moreover, NanoCPTs presented a negative surface potential with a zeta potential of −10.5 mV (Table 1), that may cause by hydroxyl of CPT molecules explored on the surface of NanoCPTs.

Furthermore, in situ polymerization was initiated on the surface of NanoCPTs using the OP-AC surface ligand, and by utilizing BIS and BAC crosslinkers, both the nonresponsive CPT-NCs-A and the reduction-responsive CPT-NCs-B were successfully prepared. Figure 2B,C show TEM images of CPT-NCs-A and CPT-NCs-B. Both CPT-NCs exhibited a spherical core-shell structure, comprising a dark CPT particle core and a gray polymer shell, thus suggesting that the polymer layer was successfully coated on the surface of the NanoCPTs by the in situ polymerization. As compared to NanoCPTs, the particle size of CPT-NCs was significantly increased, and the average hydrodynamic sizes of CPT-NCs-A and CPT-NCs-B were determined to be approximately 245.6 nm and 343.4 nm, respectively (Figure 2E,F and Table 1). After in situ polymerization, significant charge conversions from −10.5 mV of NanoCPT to +17.8 and +13.9 mV were respectively noted for CPT-NCs-A and CPT-NCs-B, demonstrating that neutral polyacrylamide (PAAm) was coated on the surface of NanoCPT. Taken together, the significant particle size increase and surface charge conversion indicated that CPT-NCs had been successfully prepared by in situ polymerization.

### 3.2. In Vitro CPT Release from the CPT-NCs

**Figure 3:**
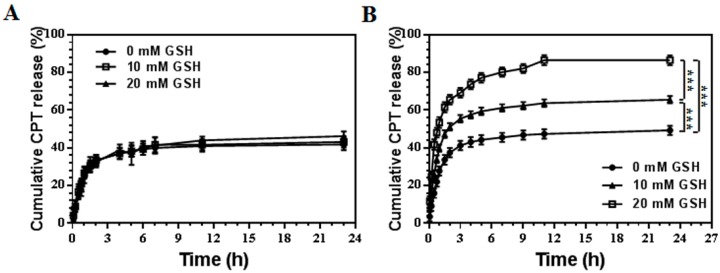
In vitro release kinetics of camptothecin (CPT) from (**A**) CPT-NCs-A and (**B**) CPT-NCs-B in a phosphate-buffered saline (PBS) buffer at a pH = 7.4 at different glutathione (GSH) concentrations. Data represent means ± SD (*n* = 3), and *** *p* < 0.001.

The reduction-responsive nanocarriers containing disulfide bonds, which can be triggered to burst release the payload drugs by collapsing the original structures by dividing the disulfide bonds under high level of GSH. The dialysis method was employed to test the drug release of both CPT-NCs-A (containing nondegradable crosslinker BIS) and CPT-NCs-B (containing reductive-degradable crosslinker BAC) in vitro. We investigated the cumulative drug release based on the UV absorption intensity of the solution containing released CPT at various reducing agent (GSH) concentrations of 0, 10, and 20 mM, at 37 °C. As shown in Figure 3A, the CPT release behavior of CPT-NCs-A at various GSH concentrations was almost the same as that without GSH, thus suggesting that CPT-NCs-A had no reduction-responsive drug release behaviors. In contrast, it was found that the drug release profiles of CPT-NCs-B were strongly dependent on the concentration of GSH (Figure 3B). The presence of GSH can divide the disulfide bond in the polymer layer of CPT-NCs-B, thus resulting in a burst release of payload CPT. At a GSH concentration of 10 mM, approximately 60% of CPT was released from CPT-NCs-B during the incubation period of 24 h. After the GSH concentration increased to 20 mM, a much higher cumulative released amount of approximately 85% was observed during the same incubation period. These results strongly suggested that high level of reducing agent of GSH could trigger significantly higher and faster drug release from CPT-NCs-B compared to normal physiological conditions.

### 3.3. Cellular Uptake and Internalization Pathway of the CPT-NCs

**Figure 4:**
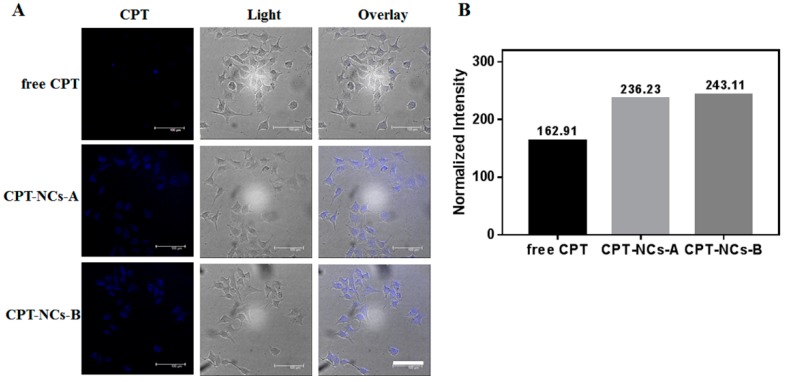
(**A**) confocal laser scanning microscope (CLSM) of SCC-15 cells after treatment with free CPT, CPT-NCs-A, and CPT-NCs-B for 4 h. Scale bar = 100 µm; (**B**) The quantitative fluorescence intensity of squamous cell carcinoma (SCC-15) cells after treatment with free CPT, CPT-NCs-A, and CPT-NCs-B for 4 h by Image-Pro Plus.

We subsequently studied the cellular uptake of free CPT, CPT-NCs-A and CPT-NCs-B using CLSM in SCC-15 cells. The cells were observed on CLSM after incubation with testing samples for 4 h. As shown in Figure 4A, the cells co-incubated with free CPT exhibited no obvious fluorescent signal of CPT. However, blue fluorescent CPT signals were distinctly visible in the cells co-incubated with both CPT-NCs-A and CPT-NCs-B. The quantitative fluorescence intensity is shown in Figure 4B; as compared to free CPT, SCC-15 cells co-incubated with both CPT-NCs-A and CPT-NCs-B exhibited higher intensity, which suggested that CPT-NCs could be efficiently internalized by the cells.

**Figure 5:**
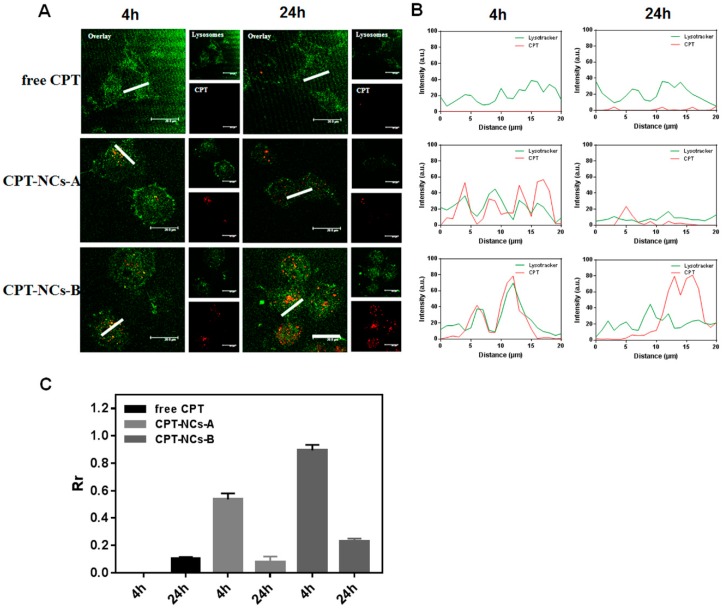
(**A**) CLSM of LysoTracker Green staining (green = endosomes or lysosomes, red = CPT) of SCC-15 cells after treatment with free CPT, CPT-NCs-A, and CPT-NCs-B for 4 h and 24 h. Scale bar = 20 µm; (**B**) Co-localization analysis of SCC-15 cells after treatment with free CPT, CPT-NCs-A, and CPT-NCs-B for 4 h and 24 h by Image-Pro Plus; (**C**) Pearson’s correlation coefficient of the red/green fluorescence in SCC-15 cells after treatment with free CPT, CPT-NCs-A, and CPT-NCs-B for 4 h and 24 h (*n* = 3).

Furthermore, the cellular trafficking mechanism of the internalized CPT-NCs was investigated by tracking the CPT fluorescence at different time points (4 h and 24 h), and by monitoring the co-localization using LysoTracker Green DNA-26 probe for late endosomes and lysosomes using CLSM. As shown in Figure 5A, the yellow fluorescent signal, which was the end outcome of the overlap of CPT (red) and lysosome signals (green), was observed after 4 h of incubation, confirming that CPT-NCs were trafficked into endosomes/lysosomes via the endocytosis pathway. After 24 h of incubation, the degree of co-localization of CPT and endosome/lysosome signals decreased, and single red fluorescent signal of CPT was detected, strongly suggesting that most of the CPT-NCs were delivered into the cytoplasm. Figure 5B shows the corresponding fluorescence intensity profiles across the white line (as marked in the CLSM images) by Image-Pro Plus. For the cells incubated with CPT-NCs for 4 h, the overlapping of red CPT-NCs fluorescence with green endosomes/lysosomes fluorescence was observed, suggesting that most of the CPT-NCs remained in the endosomes/lysosomes. However, after 24 h of incubation, the red CPT-NCs fluorescence was outside the area of the green endosomes/lysosomes fluorescence, demonstrating that the CPT-NCs escaped from endosomes/lysosomes at this time point. Furthermore, the Pearson’s correlation coefficients (Rr) were used to quantitatively analyze the correlation between red CPT-NCs fluorescence and green endosomes/lysosomes fluorescence [28]. As shown in Figure 5C, the Rr values of 0.53 and 0.07 were observed for CPT-NCs-A incubated for 4 h and 24 h, respectively, demonstrating that there is significant difference in the co-localization effect of CPT-NCs-A and endosomes/lysosomes at various incubation time. Moreover, the Rr values of 0.89 and 0.22 were observed for CPT-NCs-B incubated for 4 h and 24 h, respectively, that also proved significant separation of CPT-NCs-B from endosomes/lysosomes with increasing of incubation time. Taken together, these results from CLSM images validated that CPT-NCs were indeed internalized by the cells and could escape from endosomes/lysosomes to reach the desired destination [31].

### 3.4. In Vitro Cytotoxicity Assay of the CPT-NCs

**Figure 6:**
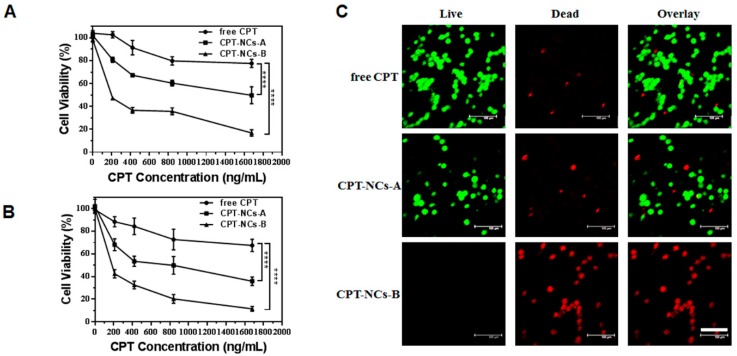
3-(4,5-dimethylthiazol-2-yl)-2,5-diphenyltetrazolium bromide (MTT) assay of SCC-15 cells after treatment with free CPT, CPT-NCs-A, and CPT-NCs-B for 24 h (**A**) and 48 h (**B**). Data represent means ± SD (*n* = 3), and **** *p* < 0.0001. (**B**) CLSM of live/dead staining (green = live cells, red = dead cells) of SCC-15 cells after treatment with free CPT, CPT-NCs-A, and CPT-NCs-B for 24 h. Scale bar = 100 µm.

After determining that CPT-NCs were delivered into the cytoplasm, we compared the induced cytotoxicity of SCC-15 cells after treatment with free CPT, CPT-NCs-A, and CPT-NCs-B for 24 h and 48 h using the MTT assay. As displayed in Figure 6A,B, free CPT exhibited no significant cytotoxicity owing to the fact that it cannot be internalized by the cells. Furthermore, in comparison to CPT-NCs-A, the cells treated with CPT-NCs-B displayed a higher cytotoxicity with 16.8% and 11.5% living cells at the highest CPT concentration of 1.68 µg/mL for 24 h and 48 h incubation, respectively. The half maximal inhibitory concentration (IC_50_) value of CPT-NCs-B at 24 h of incubation was approximately 183.5 ng/mL, while the IC_50_ value of CPT-NCs-A was 1591 ng/mL. The increased cytotoxicity of CPT-NCs-B was attributed to the enhanced CPT release, which was mediated by the degradation of the polymer layer of CPT-NCs-B in the reducing environment of the cell cytoplasm [32].

Moreover, we used live/dead assays to visually distinguish between live and dead cells after incubating the cells with the testing samples. The live and dead cells were stained with ethidium homodimer-1 (green) and calcein AM (red), respectively. From the CLSM images (Figure 6C), the cells treated with CPT-NCs-B resulted in a lower viability as compared to the cases where free CPT and CPT-NCs-A were used at the CPT concentration of 1.68 µg/mL. This was evidenced by the fact that most of the cells elicited red fluorescence, thus confirming the finding that CPT-NCs-B induced higher cytotoxicity.

### 3.5. In Vitro Apoptosis Assay of the CPT-NCs

**Figure 7:**
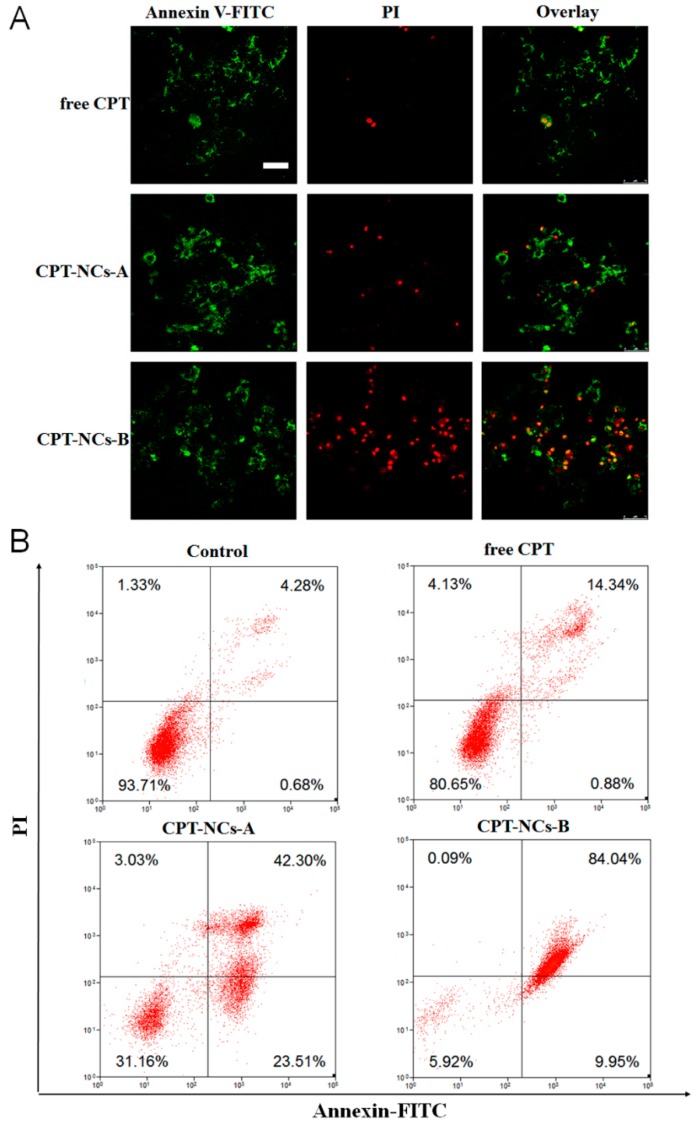
(**A**) CLSM of Annexin V-fluorescein isothiocyanate isomer (FITC)/propidium iodide (PI) double staining (green = early apoptotic cells, red = late apoptotic cells) of SCC-15 cells after treatment with free CPT, CPT-NCs-A, and CPT-NCs-B. Scale bar = 75 µm; (**B**) Flow cytometry (FCM) analysis of Annexin V-FITC/PI double staining of SCC-15 cells after treatment with PBS, free CPT, CPT-NCs-A, and CPT-NCs-B.

An Annexin-V/PI double-staining assay was used to assess the apoptotic or necrotic inducing capability of the CPT-NCs, which was imaged by CLSM and quantified by FCM analysis in Figure 7. As displayed in Figure 7A, the increased numbers of late apoptotic or necrotic cells induced by CPT-NCs-B were evidenced by the increased red fluorescence of labeled cells compared to the cases where free CPT and CPT-NCs-As were used. Figure 7B shows that the cells treated with CPT-NCs-B presented a 84.04% late apoptotic or necrotic ratio, which was significantly higher than those for the control, free CPT, and CPT-NCs-A groups, which yielded ratios of 4.28%, 14.34%, and 42.30%, respectively. Taken together, the results of the Annexin-V/PI double-staining assay validated that reduction-responsive CPT-NCs-B had an outstanding ability to induce cell apoptosis or necrosis, which may be caused by the effective CPT release rate in the cell cytoplasm.

## 4. Conclusions

In conclusion, we developed a smart reduction-responsive CPT nanocapsule by combining nanoprecipitation and in situ polymerization techniques with the use of a polymerized surface ligand. CPT nanocapsules presented spherical core-shell structures with a drug particle core and a polymer shell. The drug release studies proved that CPT nanocapsules that contained disulfide bond crosslinkers exhibited sensitive drug release behaviors at high-levels of GSH, which could lead to the rapid release of the encapsulated CPT in the cell cytoplasm. In addition, the cellular uptake, cytotoxic, and apoptotic investigations demonstrated that CPT nanocapsules could be efficiently taken up by the cells via the endocytosis pathway, and thus achieved an outstanding cytotoxic response against cancer cells in squamous cell carcinoma. Finally, we believe that this synthetic strategy of drug–polymer nanocapsules offers a new approach for the design of smart responsive nanomedicine drugs using various functional monomers and stimuli-responsive crosslinkers for the treatment of multiple other diseases.

## Figures and Tables

**Table 1:** Hydrodynamic sizes and zeta potentials of NanoCPTs and CPT-NCs.

Name	NanoCPTs	CPT-NCs-A	CPT-NCs-B
Size (d. nm)	32.7	245.6	232.3
Zeta potential (mV)	−10.5	+17.8	+13.9

## References

[1] Hu X., Li F., Wang S., Xia F., Ling D. (2018). Biological stimulus-driven assembly/disassembly of functional nanoparticles for targeted delivery, controlled activation, and bioelimination. Adv. Healthc. Mater..

[2] Guan L., Rizzello L., Battaglia G. (2015). Polymersomes and their applications in cancer delivery and therapy. Nanomedicine.

[3] Haidar Z.S. (2010). Bio-inspired/-functional colloidal core-shell polymeric-based nanosystems: Technology promise in tissue engineering, bioimaging and nanomedicine. Polymers.

[4] Wang R., Billone P.S., Mullett W.M. (2013). Nanomedicine in action: An overview of cancer nanomedicine on the market and in clinical trials. J. Nanomater..

[5] Jana D., Jana C., Vojtech A., Marketa R., Tomas E., Jaromir H., Rene K. (2013). Nanocarriers for anticancer drugs—New trends in nanomedicine. Curr. Drug Metab..

[6] Onaca O., Enea R., Hughes D.W., Meier W. (2009). Stimuli-responsive polymersomes as nanocarriers for drug and gene delivery. Macromol. Biosci..

[7] Han S., Liu Y., Nie X., Xu Q., Jiao F., Li W., Zhao Y., Wu Y., Chen C. (2012). Efficient delivery of antitumor drug to the nuclei of tumor cells by amphiphilic biodegradable poly(l-aspartic acid-co-lactic acid)/dppe co-polymer nanoparticles. Small.

[8] Tan C., Wang Y., Fan W. (2013). Exploring polymeric micelles for improved delivery of anticancer agents: Recent developments in preclinical studies. Pharmaceutics.

[9] Jay P.J., Wubeante Y.A., Neeraj K. (2011). Self-assembling polymers as polymersomes for drug delivery. Curr. Pharm. Des..

[10] Zhang N., Wardwell R.P., Bader A.R. (2013). Polysaccharide-based micelles for drug delivery. Pharmaceutics.

[11] Sun Q., Radosz M., Shen Y. (2012). Challenges in design of translational nanocarriers. J. Control. Release.

[12] Lee J.S., Feijen J. (2012). Polymersomes for drug delivery: Design, formation and characterization. J. Control. Release.

[13] Ryu J.-H., Chacko R.T., Jiwpanich S., Bickerton S., Babu R.P., Thayumanavan S. (2010). Self-cross-linked polymer nanogels: A versatile nanoscopic drug delivery platform. J. Am Chem. Soc..

[14] Städler B., Price A.D., Zelikin A.N. (2011). A critical look at multilayered polymer capsules in biomedicine: Drug carriers, artificial organelles, and cell mimics. Adv. Funct. Mater..

[15] Hinton T.M., Monaghan P., Green D., Kooijmans S.A.A., Shi S., Breheney K., Tizard M., Nicolazzo J.A., Zelikin A.N., Wark K. (2012). Biodistribution of polymer hydrogel capsules for the delivery of therapeutics. Acta Biomater..

[16] Wang Y., Yan Y., Cui J., Hosta-Rigau L., Heath J.K., Nice E.C., Caruso F. (2010). Encapsulation of water-insoluble drugs in polymer capsules prepared using mesoporous silica templates for intracellular drug delivery. Adv. Mater..

[17] Guo Y., Xu G., Yang X., Ruan K., Ma T., Zhang Q., Gu J., Wu Y., Liu H., Guo Z. (2018). Significantly enhanced and precisely modeled thermal conductivity in polyimide nanocomposites with chemically modified graphene via in situ polymerization and electrospinning-hot press technology. J. Mater. Chem. C.

[18] Wang Y., Wang Y., Hosono E., Wang K., Zhou H. (2008). The design of a LiFePO_4_/carbon nanocomposite with a core–shell structure and its synthesis by an in situ polymerization restriction method. Angew. Chem. Int. Ed..

[19] Hu Z., Liu C. (2012). Polyethylene/graphite oxide nanocomposites obtained by in situ polymerization using modified graphite oxide-supported metallocene catalysts. J. Polym. Res..

[20] Jia X., Wang L., Du J. (2018). In situ polymerization on biomacromolecules for nanomedicines. Nano Res..

[21] Mura S., Nicolas J., Couvreur P. (2013). Stimuli-responsive nanocarriers for drug delivery. Nat. Mater..

[22] Sun C.Y., Liu Y., Du J.Z., Cao Z.T., Xu C.F., Wang J. (2015). Facile generation of Tumor-pH-Labile linkage-bridged block copolymers for chemotherapeutic delivery. Angew. Chem. Int. Ed..

[23] Zhang J., Li Y., Wang J., Qi S., Song X., Tao C., Le Y., Wen N., Chen J. (2017). Dual redox-responsive PEG-PPS-cRGD self-crosslinked nanocapsules for targeted chemotherapy of squamous cell carcinoma. RSC Adv..

[24] Zhu L., Kate P., Torchilin V.P. (2012). Matrix metalloprotease 2-responsive multifunctional liposomal nanocarrier for enhanced tumor targeting. ACS Nano.

[25] Lee C.-S., Na K. (2014). Photochemically triggered cytosolic drug delivery using pH-responsive hyaluronic acid nanoparticles for light-induced cancer therapy. Biomacromolecules.

[26] Zhao M., Biswas A., Hu B., Joo K.-I., Wang P., Gu Z., Tang Y. (2011). Redox-responsive nanocapsules for intracellular protein delivery. Biomaterials.

[27] Li J., Huo M., Wang J., Zhou J., Mohammad J.M., Zhang Y., Zhu Q., Waddad A.Y., Zhang Q. (2012). Redox-sensitive micelles self-assembled from amphiphilic hyaluronic acid-deoxycholic acid conjugates for targeted intracellular delivery of paclitaxel. Biomaterials.

[28] Wang Y., Wei G., Zhang X., Xu F., Xiong X., Zhou S. (2017). A step-by-step multiple stimuli-responsive nanoplatform for enhancing combined chemo-photodynamic therapy. Adv. Mater..

[29] Han S.-S., Li Z.-Y., Zhu J.-Y., Han K., Zeng Z.-Y., Hong W., Li W.-X., Jia H.-Z., Liu Y., Zhuo R.-X. (2015). Dual-Ph sensitive charge-reversal polypeptide micelles for tumor-triggered targeting uptake and nuclear drug delivery. Small.

[30] Jiang G., Liu C., Liu X., Zhang G., Yang M., Liu F. (2009). Construction and properties of hydrophobic association hydrogels with high mechanical strength and reforming capability. Macromol. Mater. Eng..

[31] Jiang T., Mo R., Bellotti A., Zhou J., Gu Z. (2014). Gel-liposome-mediated co-delivery of anticancer membrane-associated proteins and small-molecule drugs for enhanced therapeutic efficacy. Adv. Funct. Mater..

[32] Chen D., Zhang G., Li R., Guan M., Wang X., Zou T., Zhang Y., Wang C., Shu C., Hong H. (2018). Biodegradable, hydrogen peroxide, and glutathione dual responsive nanoparticles for potential programmable paclitaxel release. J. Am. Chem. Soc..

